# Genome-wide comparative analysis of the nucleotide-binding site-encoding genes in four *Ipomoea* species

**DOI:** 10.3389/fpls.2022.960723

**Published:** 2022-08-18

**Authors:** Zengzhi Si, Lianjun Wang, Yake Qiao, Rajib Roychowdhury, Zhixin Ji, Kai Zhang, Jinling Han

**Affiliations:** ^1^Hebei Key Laboratory of Crop Stress Biology, Hebei Normal University of Science and Technology, Qinhuangdao, China; ^2^Institute of Food Crops, Hubei Academy of Agricultural Sciences, Wuhan, China; ^3^Department of Plant Pathology and Weed Research, Institute of Plant Protection, Agricultural Research Organization (ARO)–Volcani Center, Rishon LeZion, Israel

**Keywords:** NBS-encoding gene, *Ipomoea* species, phylogenetic relationship, chromosomal location, syntenic analysis, expression analysis, disease resistance

## Abstract

The nucleotide-binding site (NBS)-encoding gene is a major type of resistance (R) gene, and its diverse evolutionary patterns were analyzed in different angiosperm lineages. Until now, no comparative studies have been done on the NBS encoding genes in *Ipomoea* species. In this study, various numbers of NBS-encoding genes were identified across the whole genome of sweet potato (*Ipomoea batatas*) (#889), *Ipomoea trifida* (#554), *Ipomoea triloba* (#571), and *Ipomoea nil* (#757). Gene analysis showed that the CN-type and N-type were more common than the other types of NBS-encoding genes. The phylogenetic analysis revealed that the NBS-encoding genes formed three monophyletic clades: CNL, TNL, and RNL, which were distinguished by amino acid motifs. The distribution of the NBS-encoding genes among the chromosomes was non-random and uneven; 83.13, 76.71, 90.37, and 86.39% of the genes occurred in clusters in sweet potato, *I. trifida*, *I. triloba*, and *I. nil*, respectively. The duplication pattern analysis reveals the presence of higher segmentally duplicated genes in sweet potatoes than tandemly duplicated ones. The opposite trend was found for the other three species. A total of 201 NBS-encoding orthologous genes were found to form synteny gene pairs between any two of the four *Ipomea* species, suggesting that each of the synteny gene pairs was derived from a common ancestor. The gene expression patterns were acquired by analyzing using the published datasets. To explore the candidate resistant genes in sweet potato, transcriptome analysis has been carried out using two resistant (JK20 and JK274) and susceptible cultivars (Tengfei and Santiandao) of sweet potato for stem nematodes and *Ceratocystis fimbriata* pathogen, respectively. A total of 11 differentially expressed genes (DEGs) were found in Tengfei and JK20 for stem nematodes and 19 DEGs in Santiandao and JK274 for *C. fimbriata*. Moreover, six DEGs were further selected for quantitative reverse-transcription polymerase chain reaction (qRT-PCR) analysis, and the results were consistent with the transcriptome analysis. The results may provide new insights into the evolution of NBS-encoding genes in the *Ipomoea* genome and contribute to the future molecular breeding of sweet potatoes.

## Introduction

Throughout their life cycle, the land plants are more prone to diseases caused by a wide range of pathogens, including fungus, bacteria, nematodes, virus, etc. Through the evolutionary process, plants have developed an efficient immune system, which is composed of pattern-triggered immunity (PTI) and effector-triggered immunity (ETI) (Dodds and Rathjen, 2010). The PTI, considered as the first line of defense by responding to pathogen-associated molecular patterns (PAMPs), was recognized through the extracellular receptors. Due to the host-pathogen co-evolution, the ever-evolving pathogen can easily break the PTI barrier of the host plant. ETI, the second layer of the defense system of the host plant, is activated *via* perceiving the signals from pathogen-secreted effector proteins which directly or indirectly trigger the sequential activation of disease resistance genes (*R*-genes) (Dodds and Rathjen, 2010). Since the isolation and cloning of the first plant disease resistance gene, *Hml* (round spot resistance gene) (Johal and Briggs, 1992), researchers have isolated and cloned more than 300 *R*-genes from various plants. The majority of the *R*-genes encode nucleotide-binding site (NBS) (Liu et al., 2021).

Nucleotide-binding site-encoding *R*-genes constitute a large gene family and are commonly found in plant genomes. Based on the N-terminal domain, the NBS-encoding proteins were divided into (i) *Drosophila* Toll and the mammalian Interleukin-1 receptor (TIR)-NBS-leucine-rich repeat (LRR) (TNL), (ii) coil-coil (CC)-NBS-LRR (CNL), and (iii) resistance to powdery mildew (RPW8)-NBS-LRR (RNL) (Shao et al., 2014). Most TNL and CNL proteins function as pathogen detectors, either directly interacting with pathogen effectors or monitoring the state changes of host proteins targeted by effectors (Kourelis and van der Hoorn, 2018; Zhou et al., 2020). The protein encoded by the RNL gene is “helper” nucleotide-binding leucine-rich-repeat (NLR), which is involved in downstream signal transduction of TNL and CNL proteins (Kourelis and van der Hoorn, 2018; Wang et al., 2020). In the green algae, NBS-LRR gene has a very low copy number and often lost (Shao et al., 2019). During the process of evolution of land plants, NLR genes are significantly increased it copy number in plant genomes. For example, angiosperm genome usually has dozens to hundreds of NLR genes, most of which belong to TNL and CNL subfamilies (Liu et al., 2021).

As many plant genome sequences have become available, the first comprehensive study of genome-wide evolutionary analysis of the NBS-coding genes has been carried out in *Arabidopsis* (Meyers et al., 2003), and presently applied to other plants as well. In addition, comparative genomic studies of NBS-coding gene families have been carried out in related species that show different evolutionary patterns. For example, frequent gene loss and limited gene duplication resulted in a small number of genes encoding NBS in the three Cucurbitaceae species (Lin et al., 2013). Among the three Solanaceae crops, pepper showed a “contraction” mode, tomato showed “first expansion and then contraction” modes, and potato showed a “continuous expansion” mode (Luo et al., 2012). The number of NLR genes may also vary greatly in different genomes of the same family: the difference in the number of genes is approximately 2–6 times (Luo et al., 2012; Shao et al., 2014; Qian et al., 2017). A recent study demonstrates that the number of NLR genes varies by as much as 20-fold in Orchids (Xue et al., 2020). *Phalaenopsis equestris* and *Dendrobium catenatum* exhibited an evolutionary pattern of “early contraction to recent expansion,” while *Gastrodia elata* and *Apostasia shenzhenica* showed an evolutionary pattern of “contraction” (Xue et al., 2020).

*Ipomoea* is the largest genus of Convolvulaceae and contains 600–700 species (Austin and Huáman, 1996). *Ipomoea* species are globally distributed with agricultural and industrial importance (Liu, 2017; Morita and Hoshino, 2018; Sun et al., 2022). Sweet potato, *Ipomoea batatas* (L.) Lam. (2*n* = 6*x* = 90), ranked as the seventh most important global crop, is not only an important food and fodder crop but also an important industrial raw material for new energy plant (Liu, 2011, 2017). The diploids, *Ipomoea trifida* and *Ipomoea triloba*, are the closest wild relatives of hexaploid sweet potato (Hirakawa et al., 2015; Li et al., 2019). Compared to the hexaploid species, the diploid wild relatives are more resistant to diseases and are tolerant to abiotic stresses (Kowyama et al., 1996). These wild species are much important for the genetic improvement of sweet potato and associated evolutionary analysis. As both of these wild species have smaller genomes, they act as ideal model species for studying the genome evolution in *Ipomoea*. *Ipomoea nil* (L.) Roth. (2*n* = 2*x* = 30) is cultivated as an ornamental plant due to its diverse flower color patterns (Hoshino et al., 2016; Morita and Hoshino, 2018), and it is usually used as a rootstock for sweet potato grafting-mediated breeding to induce flowering and floral variations (Wei et al., 2019).

In spite of the importance of *Ipomoea* species, very limited genetics and genomics studies has been done so far, especially in the sweet potato which possesses a very complex genomic nature (Ozias-Akins and Jarret, 1994). In recent years, haplotype-based genome assembly of sweet potato was carried out that provides useful information on its complex genome (Yang et al., 2017). Based on the reference genome sequence, 315 NBS-encoded genes were identified recently in sweet potato (Si et al., 2021). The number of NBS-coding genes in sweet potato could be underestimated due to the lack of existing study and research progress (Wu et al., 2018). So far, there is no report on the comparative genomics of NBS-coding genes in *I. trifida*, *I. triloba*, and *I. nil*, and the evolutionary model of NBS-coding genes in sweet potato and its wild relatives is still unclear.

In this present study, genome-wide identification and characterization of NBS-encoding genes were performed in the four *Ipomoea* species: *I. batatas* (sweet potato), *I. trifida*, *I. triloba*, and *I. nil*. Following this, conserved motifs, phylogenetic analysis, chromosomal distribution, gene clustering, duplication pattern, syntenic analysis, and Ka/Ks analysis were conducted on the identified NBS-encoding genes to provide a comprehensive information on NBS. Then, various transcriptome data were used for expression analysis of these genes. Based on the results, several differentially expressed genes (DEGs) were further selected for quantitative reverse-transcription polymerase chain reaction (qRT-PCR) study with selected four sweet potato cultivars (lines) for the resistance response to both sweet potato stem nematodes and *Ceratocystis fimbriata* (a fungal pathogen). This study not only provides an insight into the basic information on further functional studies of NBS-encoding genes but also lays the foundation for the molecular breeding of sweet potato in the future.

## Materials and methods

### Identification and classification of the nucleotide-binding site-encoding genes

Four *Ipomoea* species, such as *I. batatas*, *I. trifida*, *I. triloba*, and *I. nil*, were used in this study. The whole genome sequences and annotated gene model for these *Ipomoea* species were retrieved from the available open access databases, like sweet potato were downloaded from the *Ipomoea* Genome Hub,^1^ *I. trifida* and *I. triloba* were downloaded from GenBank BioProject (accessions numbers PRJNA428214 and PRJNA428241), and *I. nil* were downloaded from GenBank BioProject (accession numbers BDFN01000001-BDFN01003416).

Initially the sequences were BLASTed (Ye et al., 2006) and analyzed through hidden Markov model search (HMMsearch) as described previously (Shao et al., 2014). All protein sequences were first searched for the NB-ARC domain (Pfam accession number: PF00931) using HMMsearch with default parameters. Meanwhile, the extended amino acid sequence of the NB-ARC domain was used as a query to search for all remaining protein sequences in the sweet potato genome using the BLASTP program. For quality control (QC), the threshold expectation value was set to 1.0, as described in previous research (Li et al., 2010). The gene sequences obtained from HMMsearch and BLAST methods were merged, and the redundant ones were removed. To further sequence confirmation to have the NB-ARC domain, sequences were checked in online Pfam^2^ for further analysis by setting the *E*-value up to 0.0001 (Shao et al., 2014). At this stage, those sequences devoid of NB-ARC domain were discarded. Pfam database and SMART protein motif analyses were used to confirm the candidate NBS-encoding genes that encoded TIR, RPW8, or LRR motifs. The COILS program was conducted to detect potential CC in the NBS-encoding genes, with a threshold value of 0.9 (Lupas et al., 1991).

### Identification of conserved motifs

To detect additional domains, such as TIR, RPW8, or LRR in the N-terminal domain and a variable number of LRR domains in the carboxy-terminal region, the raw TIR HMMs (PF01582), RPW8 (PF05659), and LRR (PF00560, PF07723, PF07725, PF12799, PF13306, PF13516, PF13855, and PF14580) were downloaded from Pfam and used for HMMsearch to identify and classify distinct domains of NBS-encoding gene candidates as described in the previous section. To investigate the structural motif diversity among the identified NBS genes, their protein sequences were subjected to motif analysis by the MEME SUITE with the minimum width of 6, maximum width of 20, the maximum number of motifs designed to identify 20 motifs and iterative cycles set to default (Bailey et al., 2009).

### Sequence alignment and phylogenetic analysis

The NBS domains (Ploop to MHDV) of the NBS-encoding genes were aligned using Clustal Omega (Sievers et al., 2011; Sievers and Higgins, 2018). MEGA 7.0 was used to remove genes with short or divergent NBS domains from the matrix (Kumar et al., 2016; Shao et al., 2016) as they interfered with fine alignment and phylogenetic analysis (Liu et al., 2021). Phylogenetic studies were carried out using IQ-TREE using the maximum likelihood approach (Minh et al., 2013), as previously described (Liu et al., 2021). The best-fit model of nucleotide substitution was estimated using ModelFinder (Kalyaanamoorthy et al., 2017), and the best-fit model JTT + F + R10 was chosen, and branch support values were calculated using SH-aLRT and UFBoot2 (Anisimova et al., 2011) with 1,000 bootstrap replicates, and *Streptomyces coelicolor* accession P25941 as an outgroup (Meyers et al., 2003). Thus, the obtained phylogenetic tree was finetuned in Figtree (ver.1.4.3) for visual improvement (Rambaut, 2014).

### Chromosome distribution and gene cluster identification

Using MapChart ver. 2.30 software, the NBS genes with chromosomal positions were mapped on the chromosomes of *Ipomoea* species (Voorrips, 2002). As the NBS-encoding genes were frequently arranged in clusters (Xiang et al., 2017), such clusters were identified in the *Ipomoea* species based on the previously established criteria that defined a chromosome or scaffold area as a gene cluster when two or more genes were localized in 200 kb (Holub, 2001; Xiang et al., 2017).

### Duplication pattern analysis of the nucleotide-binding site-encoding genes

MCScanX software was used to search for potential duplicated NBS-encoding genes in the *Ipomoea* genome (Wang et al., 2012). The protein sequences of *Ipomoea* species were compared against themselves using the BLASTP program with an *E*-value of 1e^–10^ and the results were incorporated along with the chromosomal position of all protein-coding genes. The hits were then classified into various types of duplication patterns including segmental, tandem, proximal, and dispersed using the default parameter set. The final output was visualized in the CIRCOS software (ver. 0.66) (Krzywinski et al., 2009).

### Syntenic analysis of nucleotide-binding site-encoding genes in the four *Ipomoea* genomes

Syntenic block in the genomes of used *Ipomoea* species was analyzed using MCScan software (Python version^3^) (Tang et al., 2008) with the default parameters as suggested by previous studies (VanBuren et al., 2018; Li et al., 2021). LAST was used to align the gene models, and hits were filtered to locate the best 1:1 syntenic blocks (pairs) and were visualized in the dot-plot script using JCVI package (Tang et al., 2008).

### Ka/Ks analysis of duplicated and syntenic nucleotide-binding site-encoding genes

The non-synonymous substitution (Ka) to synonymous substitution (Ks) [Ka/Ks] were calculated for both duplicated and syntenic NBS-encoding gene pairs of presently investigated four *Ipomoea* species. The nucleotide coding sequences (CDSs) of each NBS-encoding gene pair were aligned by Clustal Omega (Sievers et al., 2011; Sievers and Higgins, 2018), and the values of Ka, Ks, and Ka/Ks ratio were calculated by TBtools (Chen C. et al., 2020).

### Expression profile of nucleotide-binding site-encoding genes of the four *Ipomoea* genome

For expression profile analysis of the NBS-encoding genes in the four *Ipomoea* genomes, two bio project datasets (PRJNA511028 for *I. batatas* and PRJDB4356 for *I. nil*) were obtained from NCBI database, and the gene expressional information (fragments per kilobase of exon model per million mapped fragments, FPKM) of *I. trifida* and *I. triloba* was acquired from the sweet potato Genomics Resource.^4^ The sweet potato bio project dataset contains different tissues like fibrous root (FR), initiative storage root (ISR), distal end (DE), proximal end (PE), root body (RB), and root stalk (RS) at different stress conditions, such as salinity, drought, abscisic acid (ABA), salicylic acid (SA), and methyl jasmonate (MeJa). *I. nil* bio project dataset also contains different tissues (root, seed coat, stem, leaf, flower, and embryo). The gene expression information of *I. trifida* and *I. trifida* also contain different tissues (flower bud, flower, callus stem, callus flower, leaf, stem, and root) under different stress/treatment conditions [beta-aminobutyric acid biotic stress experiment (TTF_BABA), cold stress at 10/°C day/night experiment (TTF_COLD), biotic stress control (TTF_BICO), benzothiadiazole S-methylester biotic stress experiment (TTF_BTHT), 6-benzylaminopurine 10 uM hormone stress experiment (TTF_BAPT), hormone control experiment (TTF_HOCO), Indole-3-acetic acid 10 uM hormone stress experiment (TTF_IAAT), gibberellic acid 50 uM hormone stress experiment (TTF_GA3T), heat stress at 35/35°C day/night experiment (TTF_HEAT), drought and salt control experiment (TTF_DSCO), heat control at 28/22°C day/night experiment (TTF_HECO), cold control at 28/22°C day/night experiment (TTF_COCO), NaCl salt stress experiment (TTF_NACL), mannitol drought stress experiment (TTF_MANN), and abscisic acid 50 uM (TTF_SA)]. Meanwhile, two of our in-house transcriptome data (unpublished) for sweet potato stem nematodes and *C. fimbriata* resistance of four sweet potato cultivars or lines (sweet potato stem nematodes susceptible cultivar, “Tengfei,” sweet potato stem nematodes resistant line, “JK20,” *C. fimbriata* susceptible cultivar, “Santiandao” and *C. fimbriata* resistant line, “JK274”) were used. After removing the low-quality reads and adaptor trimming by TrimGalore software (trimming parameter: -q 25 –phred33 –length 36 -e 0.1), the clean ribonucleic acid (RNA)-Seq reads were aligned to the sweet potato reference genome sequences *via* Hisat2 (ver. 2.0.4) (Wen, 2017). Thereafter, aligned read counting has been done using SAMtools software (ver. 1.11) (Li et al., 2009). Then the obtained read counts were imported into DEseq2 (ver. 1.30.1) (Wen, 2017) for the analysis of DEGs. In each comparing case, reads were treated as DEGs if |log2FC|>1 and FDR ≤ 5%; thus, the mean log2FC value for each DEG was calculated. The heat map was constructed to visualize the distribution of the expression level of genes using the reads per kilobase per million (RPKM) value in MeV software (Howe et al., 2011).

### Ribonucleic acid isolation and quantitative reverse-transcription polymerase chain reaction analysis

The whole storage roots of the sweet potato cultivars were used for RNA extraction. The cultivars, Tengfei and JK20 were inoculated with stem nematodes (Gao et al., 2011) and the cultivars, Santiandao and JK142 were inoculated with *C. fimbriata* (Muramoto et al., 2012). Samples were collected at eight-time points (0 h, 6 h, 12 h, 1 day, 2 days, 4 days, and 6 days) after the inoculation. Root sample without inoculation was used as a control or mock. Total RNA of the samples was isolated using RNAprep Pure Plant Kit (Tiangen Biotech, Beijing, China) and first-strand cDNA was synthesized by Quantscript Reverse Transcriptase Kit (Tiangen Biotech, Beijing, China). The sweet potato β-actin gene (Genbank AY905538) was used as a control and was used to normalize the relative quantities of the three individual targeted DEGs based on its consistency across the different time points of each treatment of stem nematode and *C. fimbriata* (Liu et al., 2014). Three biological replicates were performed at each time point, and the gene expression changes were calculated using the 2^–Δ^ ^Δ^ *^Ct^* method for each sample (Schmittgen and Livak, 2008). The qRT-PCR was performed as described previously (Zhai et al., 2016) using the generated primers (Supplementary Table 1) through Primer-BLAST software (Ye et al., 2012).

## Results

### Identification and classification of the nucleotide-binding site-encoding genes

A total of 2,771 NBS-encoding genes were identified: 889 in *I. batata* (sweet potato), 554 in *I. trifida*, 571 in *I. triloba*, and 757 in *I. nil*, which account for 1.13, 1.25, 1.21, and 1.77% of the genes of the genomes, respectively (Table 1 and Supplementary Table 2). The proportion of NBS-encoding genes in hexaploid sweet potato was slightly lower than *I. trifida* and *I. triloba* and much lower than *I. nil*. To classify, each NBS-encoding proteins was identified based on N-terminal CC motifs, TIR domains, and RPW8 motifs, and LRRs. As shown in Table 1, a total of seven categories [coil-coil (CC)-NBS-LRR (CNL), coil-coil (CC)-NBS (CN), *Drosophila* Toll and the mammalian Interleukin-1 receptor (TIR)-NBS-leucine-rich repeat (LRR) (TNL), *Drosophila* Toll and the mammalian Interleukin-1 receptor (TIR)-NBS (TN), powdery mildew (RPW8)-NBS-LRR (RNL), powdery mildew (RPW8)-NBS (RN), NBS-LRR (NL), and NBS (N)] were identified. Among all NBS-encoding genes in the four *Ipomoea* species, the number of genes holding CC motif (1,244) was much greater than in TIR (333) or RPW8 motif (34). Among the four *Ipomoea* species, sweet potato, *I. trifida*, and *I. triloba* possessed a larger proportion of NL, CNL, N, and CN genes and a lower proportion of TNL, TN, RNL, and RN genes; while in *I. nil*, the proportion of CN genes was the highest and followed by N, CNL, TN, TNL, NL, RN, and RNL genes (Table 1 and Supplementary Table 2). For comparative analysis, the identified NBS-encoding genes were renamed by the method of “gene name_gene type” in the following analysis.

**TABLE 1:** Classification and distribution of nucleotide-binding site (NBS)-encoding genes in the four *Ipomoea* genomes.

Gene types	*Ipomoea batatas*		*I. trifida*		*I. triloba*		*I. nil*		Total	
	Number	Percentage (%)	Number	Percentage (%)	Number	Percentage (%)	Number	Percentage (%)	Number	Percentage (%)
CNL	229	25.76	173	31.23	174	30.47	66	8.72	642	23.17
CN	105	11.81	89	16.06	51	8.93	357	47.16	602	21.73
TNL	94	10.57	35	6.32	40	7.01	41	5.42	210	7.58
TN	27	3.04	18	3.25	17	2.98	61	8.06	123	4.44
RNL	10	1.12	8	1.44	12	2.10	0	0.00	30	1.08
RN	0	0.00	1	0.18	1	0.18	2	0.26	4	0.14
NL	288	32.40	141	25.45	190	33.27	19	2.51	638	23.02
N	136	15.30	89	16.06	86	15.06	211	27.87	522	18.84
Total	889	100.00	554	100.00	571	100.00	757	100.00	2771	100.00

Among the NBS genes in the four *Ipomoea* species, the average length of the protein sequences contain 829.06 amino acids (aa), ranging from 82 to 4,824aa. The average length of the protein sequences in *I. nil* was the largest (855.20aa, ranging from 131 to 4091aa), followed by sweet potato (840.17aa, ranging from 114 to 4824aa), *I. trifida* (807.53aa, ranging from 82 to 3,145aa), and *I. triloba* (797.97aa, ranging from 112 to 2,114aa). NBS genes in sweet potato were predicted to have 5.81 exons on average, ranging from 1 to 52, which was much larger than the average number of exons in *I. trifida* (3.57, ranging from 1 to 28), *I. triloba* (3.20, ranging from 1 to 10), and *I. nil* (3.94, ranging from 1 to 33) (Supplementary Table 2).

### Motif analysis of the nucleotide-binding site domain

**FIGURE 1:**
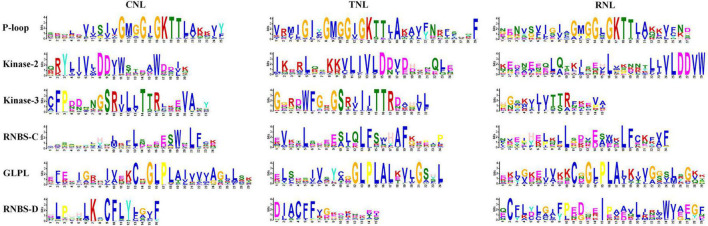
Six conserved motifs in the nucleotide-binding site (NBS) domain of the four *Ipomoea* species. The amino acids (aa) of the six conserved motifs are extracted. Larger letters indicate higher frequency.

After identifying the NBS-encoding genes, the conserved protein motifs of CNL-, TNL-, and RNL-type of NBS were analyzed. From the N-terminus to the C-terminus, six conserved motifs were detected in each subclass of the NBS domains in the four *Ipomoea* species: P-loop, Kinase-2, Kinase-3, RNBS-C, GLPL, and RNBS-D motifs (Figure 1). The P-loop, Kinase-3, RNBS-C, and GLPL motifs were more conserved than Kinase-2 and RNBS-D motifs as a whole with its aa sequences (Supplementary Table 3). In the CNL- and RNL-type of NBS, the P-loop, Kinase-3, RNBS-C, GLPL, and RNBS-D motifs were more conserved than Kinase-2; whereas in the TNL-type, the P-loop, Kinase-2, Kinase-3, RNBS-C, and GLPL motifs were more conserved than RNBS-D motifs (Supplementary Table 3).

### Phylogenetic analysis of the nucleotide-binding site-encoding genes

**FIGURE 2:**
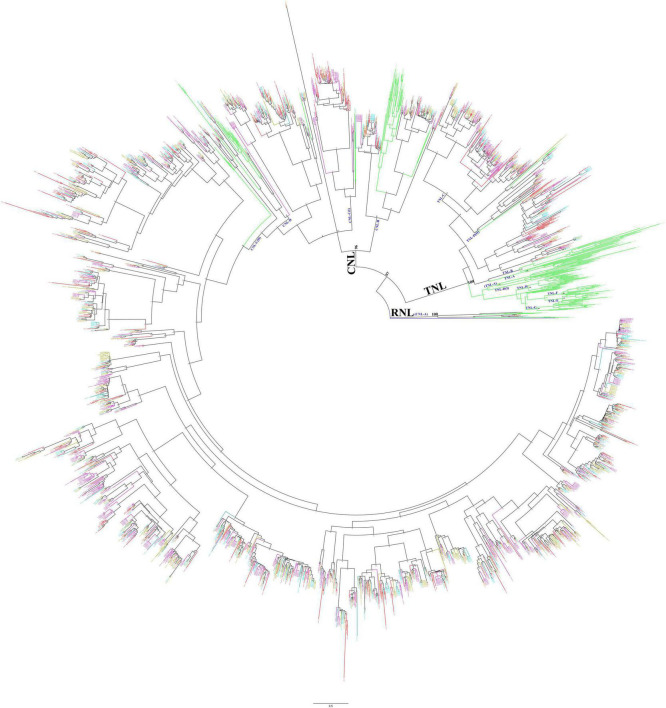
Phylogenetic relationships of the nucleotide-binding site (NBS)-encoding genes in *Ipomoea batatas*, *Ipomoea trifida*, *I. triloba*, *I. nil*, and Arabidopsis based on the amino acids (aa) of conserved NBS domains. Red, cyan-blue, olive, purple, and green lines represent the NBS-encoding genes in *I. batatas*, *I. trifida*, *I. triloba*, *I. nil*, and Arabidopsis, respectively. P25941 was used as an outgroup (marked as blue). Subclades are named as CNL-A to CNL-D and TNL-A to TNL-H.

To analyze the phylogenetic relationship of the NBS-encoding genes in *Ipomoea* species, a phylogenetic tree was constructed based on the alignment of conserved NBS domain with reference to the NBS-encoding genes of *Arabidopsis thaliana* and also from *S. coelicolor* accession, P25941 as an outgroup. After removing the sequences with incomplete and divergent NBS domains, a total of 2,218 sequences were finally used to reconstruct the evolutionary history of the NBS-encoding genes in form of phylogenetic tress (Figure 2) in which the *Ipomoea* NBS-encoding genes clustered into three independent clades: RNL, TNL, and CNL, with support values greater than 96%. The three clades exactly represent the divergence of each clade, and all genes fall into groups of corresponding classes without exception. The RNL clade was formed by the NBS-encoding genes with RPW8 domain (RNL- and RN- type genes); the TNL clade contained all of the NBS-encoding genes with TIR domain (TNL- and TN- type genes), as well as some genes from NL- and N-types, while no genes from CNL-, CN-, RNL-, and RN- types; the CNL clade contained all of the NBS-encoding genes with CC domain (CNL- and CN- type genes), as well as some genes from NL- and N-types, while there were no genes from TNL-, TN-, RNL-, and RN- types (Figure 2).

Using the NBS-encoding genes of *Arabidopsis* as a reference, the NBS-encoding genes in four *Ipomoea* species were subsequently clustered into different subclades, which were named CNL-A to CNL-D (four CNL subclades) and TNL-A to TNL-H (eight TNL subclades) (Figure 2). The interrelationship among all *Ipomoea* RNL genes within the CNL-A clade (with RPW8 domain) was very high (100% bootstrap support) which represents RNL. The CNL-B clade contained 87 *Ipomoea* NBS-encoding genes (bootstrap support = 99%): 26 from *Arabidopsis*, 19 from *I. batatas*, 14 from *I. trifida*, 14 from *I. triloba*, and 14 from *I. nil*. The CNL-C clade is partially mixed with CNL-D clade, and for better clarification, it is divided into CNL-C (I) and CNL-C (II) subclades. Both the CNL-C and CNL-D clades have weak bootstrap support. It was also found that the *Ipomoea* NBS-encoding genes with CC domain were mainly closely associated with the CNL-C (II) subclades, having a total of 1,420 genes (323 from *I. batatas*, 319 from *I. trifida*, 353 from *I. triloba*, and 425 from *I. nil*) with bootstrap support = 100% (Figure 2). The TNL group formed a genus-specific clade, except 173 *Ipomoea* genes (45 from *I. batatas*, 26 from *I. trifida*, 44 from *I. triloba*, and 58 from *I. nil*) that formed a clade with the reference to *AT1G27170* and *AT1G27180* TNL-C clade with strong support (bootstrap 92%) and 23 *Ipomoea* genes (8 from *I. batatas*, 4 from *I. trifida*, 1 from *I. triloba*, and 10 from *I. nil*) that formed a small clade with *AT5G36930* named TNL-D (II) clade with weak bootstrap support, i.e., 52% (Figure 2).

### Chromosomal location and gene cluster identification

**FIGURE 3:**
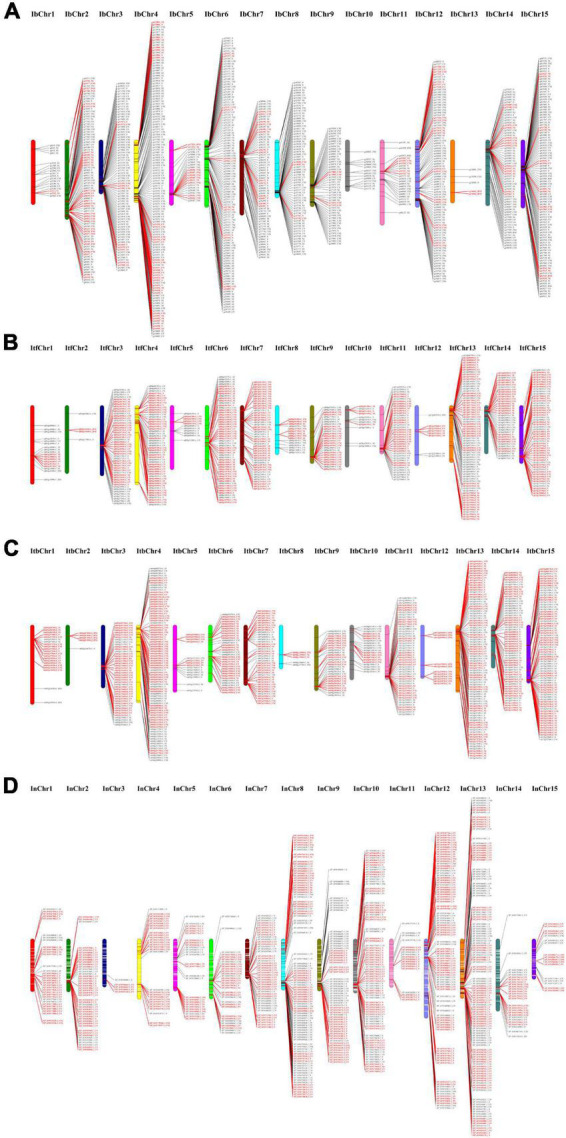
Distribution of nucleotide-binding site (NBS)-encoding genes across chromosomes. **(A)** Distribution in sweet potato chromosomes. **(B)** Distribution in *I. trifida* chromosomes. **(C)** Distribution in *I. triloba* chromosomes. **(D)** Distribution in *I. nil* chromosomes. The red color indicates the tandemly duplicated NBS-encoding genes on the chromosomal positions.

Based on the physical location of individual NBS genes, 889, 495, 571, and 731 NBS-encoding genes were mapped throughout the 15 chromosomes of *I. batatas*, *I. trifida*, *I. triloba*, and *I. nil*, respectively (Figure 3 and Supplementary Table 4). The remaining NBS genes (59 genes in *I. trifida* and 26 genes in *I. nil*) were located on the other scaffolds that had not been yet linked to a chromosome. The distribution of NBS-encoding genes was non-random across the 15 chromosomes (Figure 3 and Supplementary Table 4). For example, in sweet potato genome, the highest (#124) NBS-encoding genes were located on chr 4, whereas the lowest genes (#5) on chr 13. In *I. trifida*, chr 13 contained the highest number of NBS-encoding genes (#70), whereas only 4 NBS-encoding genes were located on chr 2. *I. triloba* chr 13 contained the highest number of NBS-encoding genes (#84), and only 5 NBS-encoding genes were located on chr 8. In *I. nil*, chr 13 contained 122 NBS-encoding genes, whereas only 5 such genes were located on chr 3 (Figure 3 and Supplementary Table 4). It is very clear that except the genome of sweet potato, chr 13 of other three *Ipomoea* species contains more NBS-encoding genes and it may have some evolutionary significance.

It was also found that about 83.13, 76.71, 90.37, and 86.39% of NBS genes were clustered in *I. batatas*, *I. trifida*, *I. triloba*, and *I. nil*, respectively. A total of 146, 96, 94, and 125 clusters containing 739, 425, 516, and 654 NBS genes in sweet potato, *I. trifida*, *I. triloba*, and *I. nil*, respectively, were identified from the *Ipomoea* genomes (Supplementary Table 4). Across the genome, the size of the clusters varied from 2 to 33, where each cluster had an average of about 5.06 (*I. batatas*), 4.43 (*I. trifida*), 5.49 (*I. triloba*), and 5.23 (*I. nil*) genes. The number of clusters genes was ranging from 2 to 115, 2 to 62, 4 to 80, and 5 to 122 across the 15 chromosomes of *I. batatas*, *I. trifida*, *I. triloba*, and *I. nil*, respectively (Supplementary Table 4). In *I. batatas*, the largest number of clusters and cluster genes was identified from chr 4 (20 clusters and 115 cluster genes), and the largest cluster containing 30 NBS-encoding genes were identified in chr 8. In *I. trifida*, the largest number of clusters and cluster genes were identified from the chr 13 (11 clusters and 62 cluster genes), and the largest cluster containing 20 NBS-encoding genes in chr 6; In *triloba*, the largest number of clusters (#15) and cluster genes (#80) were present in chr 13, and the largest cluster containing 33 NBS-encoding genes were found in chr 11. In *I. nil*, the largest number of clusters (#22) and cluster genes (#114) were positioned on chr 13, and the largest cluster containing 24 NBS-encoding genes were present in chr 13 (Supplementary Table 4).

### Duplication pattern analysis of the nucleotide-binding site-encoding genes

**FIGURE 4:**
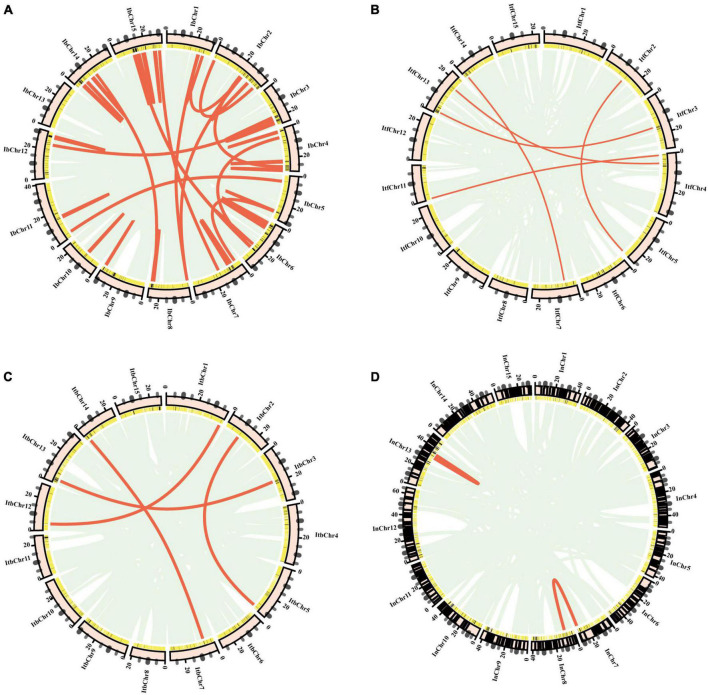
Collinear gene pairs for *I. trifida* nucleotide-binding site (NBS) genes on 15 chromosomes of four *Ipomoea* species: **(A)** *I. batatas*; **(B)** *I. trifida*; **(C)** *I. triloba*; **(D)** *I. nil*. The outer circle represents the haploid chromosomes (light pink); the second circle represents the matches of the genes with the genome of (NBS-encoding genes: black; non-NBS-encoding genes: yellow). Red lines show the collinear gene pairs for NBS-encoding genes.

Segmental and tandem duplications significantly contributed to gene family expansion in plants (Cannon et al., 2004; Kong et al., 2007; Jiang et al., 2013). Segmental duplications of multiple genes through polyploidy followed by chromosome rearrangements, and tandem duplications were characterized as multiple members of one family occurring within the same intergenic region or in neighboring intergenic regions (Zhu et al., 2014). In order to analyze the duplication pattern and adaptive evolution of NBS-encoding genes in the four *Ipomoea* species, the synteny analysis within each genome revealed a number of segmentally duplicated NBS-encoding gene pairs identified in *I. batatas* (#102), *I. trifida* (#1), *I. triloba* (#3), and *I. nil* (#4) with 94, 209, 238, and 301 tandemly duplicated gene pairs, respectively (Figure 4 and Supplementary Table 5). The proportion of segmentally duplicated genes was larger than that of tandemly duplicated ones in *I. batatas*, while this proportion of segmentally duplicated genes in other three *Ipomoea* species was much less than that of tandemly duplicated ones. The proportion of segmentally duplicated genes was larger than that of tandemly duplicated ones in sweet potato, while the proportion of segmentally duplicated genes in *I. trifida*, *I. triloba*, and *I. nil* was much smaller than that of tandemly duplicated ones. These results suggested that both segmental and tandem duplications have played important roles in NBS-encoding gene expansion in sweet potato, and the larger proportion of segmental duplications suggested their predominant role in the evolution and expansion of sweet potato NBS-encoding genes; while in *I. trifida*, *I. triloba*, and *I. nil*, tandem duplications were predominant in NBS-encoding gene expansion.

### Syntenic analysis of nucleotide-binding site-encoding genes in the genomes of *Ipomoea*

**FIGURE 5:**
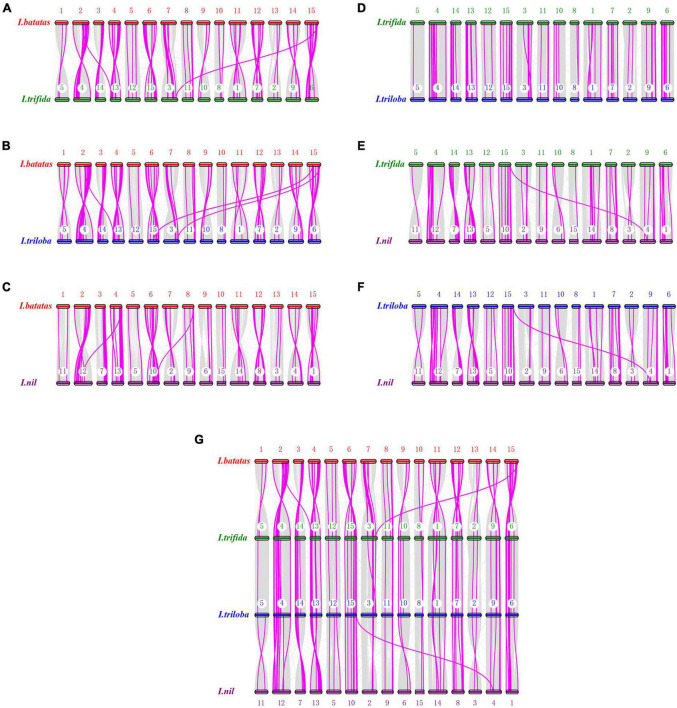
Synteny analysis of nucleotide-binding site (NBS)-encoding genes. **(A)** Synteny between sweet potato and *I. trifida*. **(B)** Synteny between sweet potato and *I. triloba*. **(C)** Synteny between sweet potato and *I. nil*. **(D)** Synteny between *I. trifida* and *I.triloba*. **(E)** Synteny between *I. trifida* and *I. nil.* **(F)** Synteny between *I.triloba* and *I.nil*. **(G)** Schematic representation of syntenic genes among sweet potato, *I. trifida*, *I. triloba*, and *I. nil*. The chromosomes of sweet potato, *I. trifida*, *I. triloba*, and *I. nil* were assigned with red, green, blue, and purple, respectively. Gray lines connect matched gene pairs, with NBS-encoding gene pairs highlighted in pink.

Analysis of genome collinearity between the four *Ipomoea* species revealed the presence of a substantial degree of collinearity with several large collinear regions (Figure 5). In 15 chromosomes of *I. batatas*, 3 were approximately collinear with those in *I. trifida* or *I. triloba*; and 6 were collinear with those in *I. nil*. Four chromosomes of *I. nil* were approximately collinear with those in *I. trifida* or *I. triloba*. All *I. trifida* chromosomes were approximately collinear with those in *I. triloba* (Figure 5).

Synteny analysis also revealed that there were 82, 81, 78, 47, 54, and 60 syntenic blocks of NBS-encoding genes containing 244, 255, 248, 174, 178, and 188 collinear NBS pairs between *I. batatas* and *I. trifida*, *I. batatas* and *I. triloba*, *I. batatas* and *I. nil*, *I. trifida*, and *I. triloba*, *I. trifida*, and *I. nil*, *I. troloba* and *I. nil* groups, respectively (Figure 5). The syntenic NBS-encoding genes had three different forms: singleton to singleton, singleton to cluster, and cluster to cluster (Figure 5). The syntenic blocks of NBS genes were consistent with the detected syntenic chromosomes from the genomes of four *Ipomoea* species, except for a few of NBS-encoding gene pairs (Figure 5 and Supplementary Table 6).

A total of 201 NBS-encoding orthologous genes (54 from *I. batatas*, 49 from *I. trifida*, 49 from *I. triloba*, and 49 from *I. nil*) can be traced to form synteny gene pairs between any two of the four *Ipomea* species (Figure 5). The orthologous gene groups consisted of almost all of the RNL-type NBS-encoding genes and a part of other types of NBS-encoding genes (Supplementary Table 7). Further analysis revealed that synteny gene pairs between *I. batatas* and the other three *Ipomea* species were mainly observed in different combination of chromosomes of one species with others (Figure 5). However, no large collinear block (containing more than 3 pairs of NBS-encoding genes and with the same order of NBS-encoding genes and other genes) was found in the NBS-encoding orthologous gene groups.

### Ka/Ks analysis of duplicated and syntenic nucleotide-binding site-encoding genes

The non-synonymous substitution (Ka) to synonymous substitution (Ks) ratio (Ka/Ks) is an informative value of positive selection. To detect whether some NBS-encoding genes are under positive selection, Ka/Ks analysis was performed on duplicated and syntenic NBS-encoding genes within or between the studied four *Ipomoea* species (Supplementary Table 8). In this present investigation, most of the duplicated and syntenic NBS-encoding genes have the Ka/Ks ratio less than 1, which indicates that the majority of these genes underwent purifying (negative) evolutionary selection. Only one pair of NBS-encoding genes possessed higher Ka/Ks ratio, i.e., 1.02 in *I. batatas*. Whereas in *I. trifida*, *I. triloba*, and *I. nil*, 12, 14, and 20 pairs of duplicated NBS-encoding genes, respectively, possessed Ka/Ks ratio greater than 1. There were 10, 19, 4, 6, 2, and 3 syntenic NBS-encoding gene pairs that possessed Ka/Ks ratio greater than 1 between *I. batatas* and *I. trifida*, *I. batatas* and *I. triloba*, *I. batatas* and *I. nil*, *I. tifida* and *I. triloba*, *I. tifida* and *I. nil*, *I. triloba* and *I. nil* combinational groups, respectively (Supplementary Table 8). These results suggest that the majority of duplicated and syntenic NBS-encoding genes were subjected to purifying selection inside the duplicated genomic elements during speciation, whereas a smaller number of such genes were subjected to positive selection.

### Expression patterns of nucleotide-binding site-encoding genes in the four *Ipomoea* genomes

A total of 692 (77.84%), 203 (36.64%), 163 (28.55%), and 321 (42.40%) NBS genes have constitutive expression patterns in *I. batatas*, *I. trifida*, *I. triloba*, and *I. nil*, respectively (Supplementary Figure 1). In *I. batatas*, the NBS-encoding genes were mainly upregulated in the tissues of FR, ISR, DE of the roots, and were downregulated in the tissues of the PE, RB, and RS. Under salinity and drought stress, tissue- specific genes expression was noticed which is not in accordance with the specific stress environment, and the NBS-encoding genes can be divided into clusters according to their expression pattern. In *I. trifida* and *I. triloba*, there is no obvious tissue-specific expressions of NBS-encoding genes, except that some of the NBS-encoding genes were non-regulated and the callus flower and callus stem possessed a similar expression pattern for several NBS-encoding genes and their different expression level under different stresses. In *I. nil*, 44 NBS-encoding genes were upregulated in the embryo, while they were downregulated in other tissues; 50 NBS-encoding genes were downregulated in the root while they were non-regulated in other tissues, and some of them were non-regulated in the rest of the tissues (Supplementary Figure 1). The expression patterns of NBS-encoding genes also showed that there is a set of genes without any detected regulatory response to different tissues or stress conditions for each of the four *Ipomoea* species. A total of 31 (3.49%), 29 (5.23%), 29 (5.08%), and 58 (7.66%) NBS genes were found to have non-regulatory response in *I. batatas*, *I. trifida*, *I. triloba*, and *I. nil*, respectively.

**FIGURE 6:**
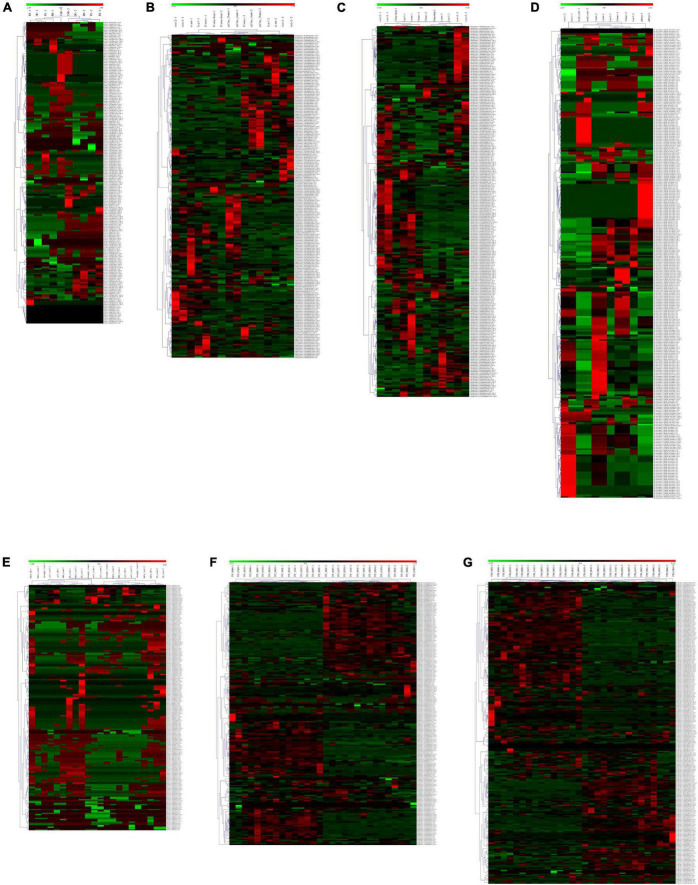
Heatmap of the compared expression profiles of duplicated nucleotide-binding site (NBS)-encoding genes in the four *Ipomoea* species. The heatmap for duplicated NBS-encoding gene pair was named as “gene1” + “^&^” + “gene2” + “duplicated patterns (-S: segmental duplication; -T: tandem duplication)” (gene1 and gene2 were duplicated gene pairs). For each tissue or stress: “-1” indicates the heatmap of “gene1”; “-2” indicates the heatmap of “gene2.” **(A)** Expression profiles of duplicated NBS-encoding genes in different sweet potato tissues: initiative storage root (ISR), distal end (DE), proximal end (PE), root body (RB), and root stalk (RS). **(B)** Expression profiles of duplicated NBS-encoding genes in different *I. trifida* tissues: the callus flower, callus stem, flower, flower bud, root 1, root 2, leaf, and stem. **(C)** Expression profiles of duplicated NBS-encoding genes in different *I. triloba* tissues: root 1, root 2, flower, flower bud, leaf, and stem. **(D)** Expression profiles of NBS-encoding genes in different *I. nil* tissues: the root, embryo, seed coat, flower, leaf, and stem. **(E)** Expression profiles of sweet potato duplicated NBS-encoding genes in response to various stress: drought, salt, methyl jasmonate (MeJa), abscisic acid (ABA), and salicylic acid (SA). **(F)** Expression profiles of *I. trifida* duplicated NBS-encoding genes in response to various stress: beta-aminobutyric acid biotic stress experiment (TTF_BABA), cold stress at 10/4°C day/night experiment (TTF_COLD), biotic stress control (TTF_BICO), benzothiadiazole S-methylester biotic stress experiment (TTF_BTHT), 6-benzylaminopurine 10 uM hormone stress experiment (TTF_BAPT), hormone control experiment (TTF_HOCO), Indole-3-acetic acid 10 uM hormone stress experiment (TTF_IAAT), gibberellic acid 50 uM hormone stress experiment (TTF_GA3T), heat stress at 35/35°C day/night experiment (TTF_HEAT), drought and salt control experiment (TTF_DSCO), heat control at 28/22°C day/night experiment (TTF_HECO), cold control at 28/22°C day/night experiment (TTF_COCO), NaCl salt stress experiment (TTF_NACL), mannitol drought stress experiment (TTF_MANN) and abscisic acid 50 uM hormone stress experiment (TTF_ABAT). **(G)** Expression profiles of *I. triloba* duplicated NBS- encoding genes in response to various stress: beta-aminobutyric acid biotic stress experiment (TTB_BABA), cold stress at 10/4°C day/night experiment (TTB_COLD), biotic stress control (TTB_BICO), benzothiadiazole S-methylester biotic stress experiment (TTB_BTHT), 6-benzylaminopurine 10 uM hormone stress experiment (TTB_BAPT), hormone control experiment (TTB_HOCO), Indole-3-acetic acid 10 uM hormone stress experiment (TTB_IAAT), gibberellic acid 50 uM hormone stress experiment (TTB_GA3T), heat stress at 35/35°C day/night experiment (TTB_HEAT), drought and salt control experiment (TTB_DSCO), heat control at 28/22°C day/night experiment (TTB_HECO), cold control at 28/22°C day/night experiment (TTB_COCO), NaCl salt stress experiment (TTB_NACL), mannitol drought stress experiment (TTB_MANN), and abscisic acid 50 uM hormone stress experiment (TTB_ABAT).

In *I. batatas*, *I. trifida*, and *I. triloba*, segmental and tandemly duplicated NBS-encoding genes tend to behave differently with each other in various tissues and stress conditions; while in *I. nil*, the tissue-specific duplicated NBS-encoding genes tended to show similar expression in the root, seed coat, stem, and embryo, while it showed different expression level in the leaf and flower (Figure 6). The results suggest that both segmentally and tandemly duplicated genes undergo functional differentiation after duplication.

**FIGURE 7:**
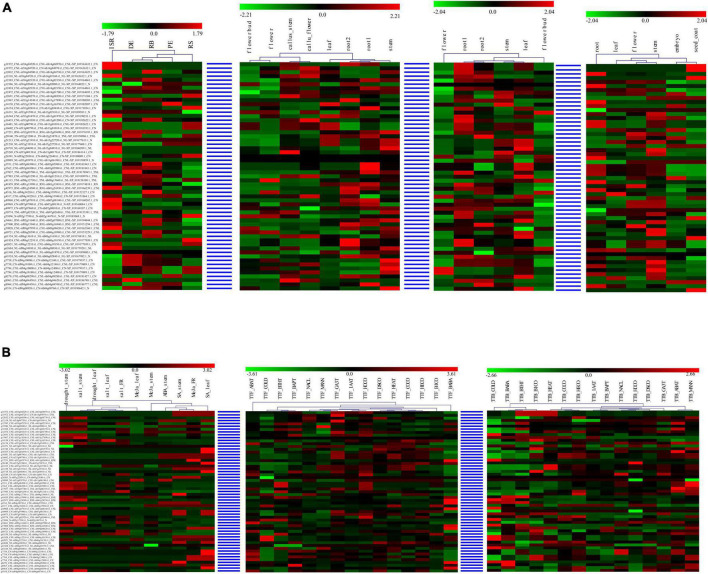
Heatmap of compared expression profiles of syntenic nucleotide-binding site (NBS)-encoding genes in the four *Ipomoea* species. The heatmap of syntenic NBS-encoding gene set was named as “sweet potato gene-” + “*I. trifida* gene-” + “*I. triloba* gene-” + “*I. nil* gene.” **(A)** Heatmap of compared expression profiles of syntenic NBS-encoding genes in the four *Ipomoea* species tissues. **(B)** Heatmap of compared expression profiles of syntenic NBS-encoding genes in the four *Ipomoea* species in response to stress.

For each syntenic NBS-encoding gene set in the four *Ipomoea* species, the expression of the genes tended to act as species-specific in various tissues and stress conditions, suggesting that syntenic NBS-encoding gene experienced species differentiation after being derived from a common ancestor (Figure 7). For example, in the syntenic NBS-encoding gene set of g11972_CNL-itf14g04520.t1_ CNL-itb14g04970.t1_CNL-XP_019163632.1_CN, *itf14g04520. t1_CNL* in the root1 of *I. trifida* and *itb14g04970.t1_CNL* in the root1 of *I. triloba* were both upregulated, while *XP_019163632.1_CN* in the root of *I. nil* was downregulated; *itf14g04520.t1_CNL* in the leaf of *I. trifida* and *XP_019163632.1_CN* in the leaf of *I. nil* were both downregulated, while *itb14g04970.t1_CNL* in the leaf of *I. triloba* was upregulated; *itf14g04520.t1_CNL* was upregulated in *I. trifida* under 50 uM gibberellic acid (GA3T) and 10 uM indole-3-acetic acid (IAAT) hormonal treatment, while the corresponding syntenic gene (*itb14g04970.t1_CNL*) in *I. triloba* was downregulated under the same hormonal condition (Figure 7).

### Transcriptome analysis of the nucleotide-binding site-encoded genes regulated by stem nematodes and *Ceratocystis fimbriata* in the cultivars of *Ipomoea batatas*

**FIGURE 8:**
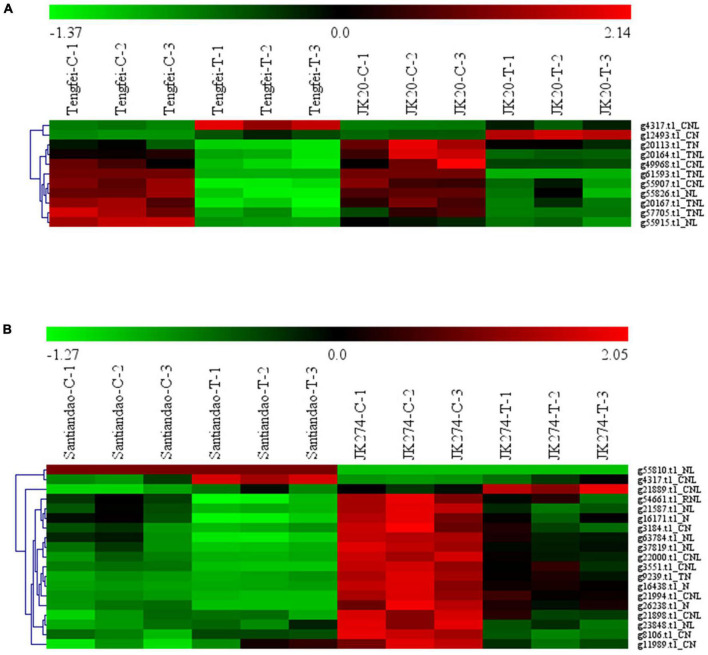
Heatmap of the expression profiles of differentially expressed genes (DEGs) in response to sweet potato stem nematodes and *Ceratocystis fimbriata* resistance. **(A)** DEGs in “Tengfei” and “JK20” under control and sweet potato stem nematodes inoculation. **(B)** DEGs in “Santiandao” and “JK274” under control and *C. fimbriata* inoculation. C, control; T, treatment.

Transcriptomic data of the *I. batatas* cultivar were assessed for stem nematode (Tengfei and JK20) and *C. fimbriata* (Santiandao and JK274) inoculation. The results revealed that 11 DEGs were found in the cultivars of stem nematodes in both control and treated conditions, and 9 of them were upregulated in the control and downregulated in the treatment. However, the transcript, *g4317.t1_CNL* was downregulated in the control of both cultivars and upregulated in the treatment of Tengfei; *g12493.t1_CN* was downregulated in the control of both cultivars, while it was upregulated in the treatment of JK20 (Figure 8).

On the other side, 19 DEGs were found in the Santiandao and JK274 under control and *C. fimbriata* inoculation treatment; 16 of them were downregulated in Santiandao under both control and treatment, while in JK274, those genes were upregulated under control and downregulated (and some non-regulated) under treatment. The transcript, *g55810.t1_NL* under both control and treatment, was upregulated in Santiandao and downregulated in JK274; *g4317.t1_CNL* was downregulated in JK274 under both control treatment, while it was downregulated under control condition and upregulated under treatment in “Santiandao”; *g21889.t1_CN* was downregulated under both control and treatment in Santiandao, while it was upregulated under control and downregulated under treatment in JK274 (Figure 8).

**FIGURE 9:**
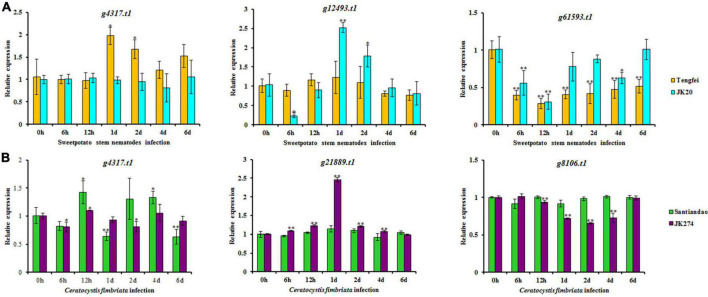
Expression analysis of nucleotide-binding site (NBS)-encoding genes on the storage roots of sweet potato cultivars or lines. **(A)** Relative expression levels of *g4317.t1*, *g12493.t1*, and *g61593.t1* in storage roots after different times of stem nematode infection. **(B)** Relative expression levels of *g4317.t1*, *g21889.t1*, and *g8106.t1* after different times of *Ceratocystis fimbriata* infection. The significance of DEG levels compared with control were denoted as ^∗^<0.05, ^∗∗^<0.01.

According to the analysis of the transcriptome data for sweet potato stem nematodes resistance, three transcripts of NBS-encoding DEGs (*g4317.t1*, *g12493.t1*, and *g61593.t1*) were selected for cross validation of gene expression level in Tengfei and JK20 under both control and different inoculation time points (Figure 9). Compared with the control condition (0 h), relative expression of *g4317.t1* was significantly induced by treatment in Tengfei even after 1–2 days of treatment, while its expression was not induced by treatment in JK20. Transcript *g12493.t1* was not induced by the treatment Tengfei, but significantly induced in JK20 under treatment. The expression of this transcript decreased in JK20 after 6 h of infection and increased in the same cultivar after 1–2 days of infection treatment. The expression level of the third transcript (*g61593.t1*) was decreased in both cultivars (Figure 9). On the other hand, three transcripts of NBS-encoding DEGs (*g4317.t1*, *g21889.t1*, and *g8106.t1*) were selected for cross validation of gene expression level in cvs. Santiandao and JK274 under both control and different inoculation time points for *C. fimbriata* (Figure 9).

Compared with the control at 0 h, the common transcript, *g4317.t1* with stem nematode experimental set was significantly induced and expressed in Santiandao after 12 h and 4 days of inoculum treatment, while its relative expression was slightly decreased by the treatment in JK274. The transcript, *g21889.t1* was not induced by the treatment in Santiandao, while its expression level was significantly induced in JK274 under same treatment condition. The same expression level of the transcript was increased in JK274 after 1 day of *C. fimbriata* infection, peaked at 1 day with 2.45-fold higher expression level than that of the control set; thereafter, the expression level declined; For *g8106.t1* by t infection, the relative transcript expression was not induced in cv. Santiandao, but slightly decreased in JK274. The transcriptomic relative expression was also consistent with the analysis of the *in silico* transcriptomic data for both stem nematode and *C. fimbriata* infection (Figure 8).

## Discussion

The most predominant type of resistance (R) genes contain nucleotide-binding sites and leucine-rich repeat (NBS-LRR) domains; their characterization is helpful for resistance improvement of plants (Liu et al., 2021). The NBS-encoding gene family has been studied in various species, including *Arabidopsis* (Meyers et al., 2003), *Oryza sativa* (Zhou et al., 2004), *Zea mays* (Cheng et al., 2012), *Triticum urartu* (Liu et al., 2017), *Gossypium hirsutum* (Shi et al., 2018), *Lagenaria siceraria* (Wang et al., 2022), and *Cucumis sativus* (Wang et al., 2022). The *Ipomoea*, with great value in the fields of industry and agriculture, is the largest genus in Convolvulaceae (Austin and Huáman, 1996; Liu, 2017; Morita and Hoshino, 2018; Sun et al., 2022). Sweet potato, together with its wild diploid ancestors (*I. trifida* and *I. triloba*), and its general rootstock (*I. nil*), are all sequenced *Ipomoea* species (Hoshino et al., 2016; Yang et al., 2017; Wu et al., 2018). However, no comprehensive and systematic research has been conducted on the NBS-encoding genes in the four important species till now. The present comparative study on genome-wide analysis of NBS-encoding genes in the four *Ipomoea* species provided new insights and useful information for *R*-gene evolution and resistance breeding.

In this study, the number of NBS-encoding genes greatly varied among the four *Ipomoea* species. The number of NBS-encoding genes varies among the four species, the number of NBS-encoding genes in the *I. batatas* (sweet potato) genome was the largest and was 1.60, 1.56, and 1.17 times greater than those in the *I. trifida*, *I. triloba*, and *I. nil*, respectively. The discrepancy of NBS-encoding genes among the three species should not be the result of ploidy variation, because *I. trifida*, *I. triloba*, and *I. nil* are all diploids and have similar chromosomal numbers, while the number of NBS-encoding genes in *I. nil* was larger than that in *I. trifida* and *I. triloba*. Moreover, there was no correlation between the NBS-encoding genes and species phylogeny or genome size has been reported by previous studies (Jacob et al., 2013; Shao et al., 2014). This may suggest that the species-specific gene loss and duplications occurred after their separation through the evolutionary lineage (Zhang et al., 2016; Liu et al., 2021). Such a phenomenon has also been reported in other closely related species (Luo et al., 2012; Shao et al., 2014; Qian et al., 2017; Xue et al., 2020; Liu et al., 2021). For example, the variation in gene number among the three major Solanaceous species, tomato (#255), potato (#447), and pepper (#306) were also quite high (Qian et al., 2017). The interspecific difference in the number of NLR genes among Orchids is 20 times higher (Xue et al., 2020). A more recent study revealed that NBS-encoding gene copy numbers differ up to 66-fold among closely related species (Liu et al., 2021). The different number of NBS-encoding genes in different species may reflect the potential disease pressures in the prevailing environments (Yang and Wang, 2016).

Our phylogenetic analysis of the NBS-encoding genes in the four *Ipomoea* species revealed three independent clades: RNL, TNL, and CNL. Moreover, the clade classification was not species-specific for the four *Ipomoea* species, which meant that species-specific gene duplication/loss events occurred after the formation of four *Ipomoea* species (Figure 2). In this study, the phylogenetic tree was constructed using the NBS-encoding genes of *A. thaliana* as a reference, and the results showed that the species-specific NBS-encoding genes appeared between *A. thaliana* and the common ancestor of the four *Ipomoea* species in each subclade (Figure 2). Compared to the reference, the common ancestor of the four *Ipomoea* species had experienced different duplications/loss events in their NBS-encoding genes. The rapid evolutionary feature of the NBS-encoding gene family caused by frequent gene duplications/loss events could elicit copy number variations (Xue et al., 2020; Zhou et al., 2020).

Although the number of NBS-encoding genes varies depending on the species, the NBS-encoding gene clustering is common in the four *Ipomoea* species. The clusters provide a reservoir of genetic variation of such genes through duplication, conversion, and diversifying selection (Ameline-Torregrosa et al., 2008). In this study, a total of 146, 96, 94, and 125 clusters containing 739, 425, 516, and 654 NBS-encoding genes in *I. batatas*, *I. trifida*, *I. triloba*, and *I. nil*, respectively, indicate non-random distribution of NBS-encoding genes across the 15 chromosomes (Figure 3 and Supplementary Table 4). The NBS-encoding gene clustering and uneven chromosomal distribution in the four *Ipomoea* species is trending similar with the previous studies on Arabidopsis (Meyers et al., 2003), *Gossypium* species (Xiang et al., 2017), *Xanthoceras sorbifolium*, *Dimocarpus longan*, and *Acer yangbiense* (Zhou et al., 2020).

The gene (genome) duplication process, segmental and tandem duplication, significantly contributed to gene family expansion and distribution of NBS-encoding gene in plants (Cannon et al., 2004; Kong et al., 2007; Jiang et al., 2013). Segmental duplications of multiple genes through polyploidy followed by chromosome rearrangements, and tandem duplications were characterized as multiple members of one family occurring within the same intergenic region or in neighboring intergenic regions (Zhu et al., 2014). In this study, segmental duplication was predominant in *I. batatas*, while tandem duplication occurred in *I. trifida*, *I. triloba*, and *I. nil* (Figures 3, 4 and Supplementary Table 5). These results suggested that both segmental and tandem duplications in *Ipomoea* genomes have played an important role in NBS-encoding gene expansion in *I. batatas*, and the larger proportion of segmental duplications suggested their predominant role in the evolution and expansion of sweet potato NBS-encoding genes; while in *I. trifida*, *I. triloba*, and *I. nil*, tandem duplications were predominant in NBS-encoding gene expansion. The previous report suggests that whole-genome triplication (WGT) occurred in an ancient ancestor of the *Ipomoea* lineage around 46.1 million years ago (Mya), much earlier than the divergence of *I. nil* from the lineage containing *I. trifida* and *I. triloba* (∼3.6 Mya), and the *I. trifida*-*I. triloba* divergence (∼2.2 Mya), the results of comparison between the genomes of *I. trifida* (or *I. triloba*) and *I. nil* limited large-scale inter-chromosomal rearrangements over the last 3.6 Mya (Wu et al., 2018), while in *I. batatas*, two recent whole-genome duplication (WGD) events occurred about 0.8 and 0.5 Mya (Yang et al., 2017). Therefore, the two recent WGD in *I. batatas* might be the reason for the discrepancies in the rate of segmental and tandem duplication. The dominance of segmental duplication in the gene family expanding in *Ipomoea* species had been reported in a previous study; it was found that segmental duplication was speculated to have a predominant role in *Ipomoea* SQUAMOSA promoter-binding protein-like genes (SPL) expansion; tandem duplication was primarily responsible for the expansion of SPL subclades IV-b and IV-c (Sun et al., 2022). Segmental and tandem duplication, along with gene losses, might bring out the uneven distributions of NBS-encoding genes in the four *Ipomoea* species (Zhou et al., 2020). The differences in the number of NBS-encoding gene clusters in the four *Ipomoea* species suggested that these species might have experienced different local duplication event after their species differentiation (Yang and Wang, 2016). The duplicated NBS- encoding genes in the four *Ipomoea* species acted in different expression profiles, suggesting that the duplicated genes have experienced functional differentiation after duplication. Such a phenomenon of variation within tandemly duplicated genes (TDGs) was reported among cultivated, non-cultivated, and wild potato genotypes in terms of bias in functional specificities, the proportion of lineage-specific clusters, diverged expression, and promoter similarities (Bonthala and Stich, 2022).

A total of 201 NBS-encoding orthologous genes from four *Ipomoea* species formed synteny gene pairs between any two of the four species (Figure 5). Similar results have been demonstrated by other closely related species. For example, in the Brassicaceae family, a number of NBS loci were identified in *Arabidopsis lyrata* (#78), *A. thaliana* (#58), *Brassica rapa* (#100), *Capsella rubella* (#52), and *Thellungiella salsuginea* (#59) (Zhang et al., 2016). These might be generated by different extents of gene duplication of the ancestor genes (Zhang et al., 2016). However, no significant collinearity was detected among the four *Ipomoea* species, which meant that these syntenic genes formed extensive rearrangement after they were derived from a common ancestor. The results of Ka/Ks analysis of the duplicated and syntenic NBS-encoding genes showed that most of the genes had a Ka/Ks ratio less than1, and a few of them harbored a Ka/Ks ratio equivalent to 1 (Supplementary Table 7). This suggests that the duplicated and syntenic NBS-encoding genes mainly undergo purifying (negative) selection within genome duplicated elements and speciation, and a very small portion shows a positive selection. Such a finding is also in accordance with the study on closely related species. For example, in five *Fragaria* species, such as *Fragaria* x *ananassa*, *Fragaria iinumae*, *Fragaria nipponica*, *Fragaria nubicola*, and *Fragaria orientalis*, most of the NBS-encoding gene pairs (98%) had Ka/Ks values less than 1, while 91 gene pairs had Ka/Ks ratios greater than 1 (Yan et al., 2018). In the grass family, the average Ka/Ks values between all orthologous groups of sorghum, rice, maize, and *Brachypodium* was 0.24, which indicate that NBS-LRR genes may undergo purifying selection after speciation in such species (Yang and Wang, 2016).

In this study, the gene expression profiles of different tissues of the four *Ipomoea* species were acquired (Supplementary Figure 1). The results showed that the four *Ipomoea* species exhibited somewhat tissue-specific expression patterns, which may be involved in the adaptation of plants to different environments (Morata and Puigdomenech, 2017). Thus, the tissue-specific expression profiling will help identify the decisive role of these genes in individual tissue in conferring resistance against pathogens (Arya et al., 2014). Based on the transcriptome data analysis on *I. batatas* cultivars in response to stem nematodes and *C. fimbriata* resistance, a significant number of DEGs were identified in specific cultivars which laid the basis of selecting NBS-encoding genes for a relative expression analysis. The expression of the genes were consistent with the transcriptome data analysis. The phenomenon that functional *R*-genes (here NBS-encoding genes) up- or downregulated by pathogen infection has been reported in other species. In rice, three homologous genes, such as *OsNPR1/NH1*, *OsNPR2/NH2*, and *OsNPR3*, were found to be induced by pathogens like rice bacterial blight (*Xanthomonas oryzae* pv. *Oryzae*) and rice blast (*Magnaporthe grisea*), and the defense chemical molecules (elicitors) like benzothiadiazole, methyl jasmonate, and ethylene, and the over-expression of *OsNPR1* not only conferred disease resistance to bacterial blight, but also enhanced herbivore susceptibility in transgenic rice plants (Yuan et al., 2007). In wheat, a set of differentially expressed transcripts (e.g., *TaGLI1*, *TaACT1*, and *TaSSI2*) were identified to be involved in glycerol and fatty acid metabolism that were upregulated in response to powdery mildew fungal infection and might contribute to glycerol-3-phosphate (G3P) and oleic acid (OA18:1) accumulation (Li et al., 2020). In poplar, 18 *R*-genes were significantly upregulated during *Marssonina brunnea* infection (Chen S. et al., 2020). Therefore, *I. batatas* stem nematodes and *C. fimbriata*-responsive NBS-encoding genes identified in the present study may be useful in studying the downstream signal transducers of pathogen sensing proteins in *Ipomoea* and other related plant species.

## Conclusion

The most predominant type of *R*-genes in plants contain NBS domains, whose characterization is helpful for improving resistance to pathogen. *Ipomoea*, being a valuable industrial and agricultural crop, no comprehensive and systematic research has been conducted on the NBS-encoding genes till date. The present comparative study on genome-wide analysis of NBS-encoding genes in the four *Ipomoea* species [*I. batatas* (sweet potato), *I. trifida*, *I. triloba*, and *I. nil*] provided new insights and useful information on *R*-gene evolution and resistance breeding.

## Data availability statement

The original contributions presented in this study are included in the article/Supplementary material, further inquiries can be directed to the corresponding author.

## Author contributions

ZS and LW: design of the study and identification of NBS-encoding genes in the four *Ipomoea* species. ZS, LW, and YQ: motif analysis. ZS, LW, and ZJ: phylogenetic analysis. ZS, KZ, and JH: chromosome location. ZS, LW, RR, and ZJ: duplication pattern analysis. ZS, LW, YQ, RR, KZ, and ZJ: syntenic analysis. ZS, KZ, RR, and JH: Ka/Ks analysis. ZS, KZ, RR, and YQ: expression profile analysis. ZS, YQ, and KZ: RT-qPCR analysis. ZS, LW, YQ, RR, KZ, ZJ, and JH: manuscript preparation. All authors contributed to the article and approved the submitted version.

## Footnotes

1
https://www.sweetpotao.com/download_genome.html

2
http://pfam.xfam.org/search#tabview=tab1

3
https://github.com/tanghaibao/jcvi/wiki/MCscan-[Python-version]

4
http://sweetpotato.uga.edu/gt4sp_download.shtml

## Supplementary material

Supplementary Figure 1 Heatmap of the expression profiles of nucleotide-binding site (NBS) genes in the four *Ipomoea* species. **(A)** Expression profiles of NBS-encoding genes in different sweet potato tissues: initiative storage root (ISR), distal end (DE), proximal end (PE), root body (RB), and root stalk (RS). **(B)** Expression profiles of NBS-encoding genes in different *I. trifida* tissues: the callus flower, callus stem, flower, flower bud, root 1, root 2, leaf, and stem. **(C)** Expression profiles of NBS-encoding genes in different *I. triloba* tissues: the root 1, root 2, flower, flower bud, leaf, and stem. **(D)** Expression profiles of NBS-encoding genes in different *I. nil* tissues: the root, embryo, seed coat, flower, leaf, and stem. **(E)** Expression profiles of sweet potato NBS-encoding genes in response to various stress: drought, salt, methyl jasmonate (MeJa), abscisic acid (ABA), and salicylic acid (SA). **(F)** Expression profiles of *I. trifida* NBS-encoding genes in response to various stress: beta-aminobutyric acid biotic stress experiment (TTF_BABA), cold stress at 10/4°C day/night experiment (TTF_COLD), biotic stress control (TTF_BICO), benzothiadiazole S-methylester biotic stress experiment (TTF_BTHT), 6-benzylaminopurine 10 uM hormone stress experiment (TTF_BAPT), hormone control experiment (TTF_HOCO), indole-3-acetic acid 10 uM hormone stress experiment (TTF_IAAT), gibberellic acid 50 uM hormone stress experiment (TTF_GA3T), heat stress at 35/35°C day/night experiment (TTF_HEAT), drought and salt control experiment (TTF_DSCO), heat control at 28/22°C day/night experiment (TTF_HECO), cold control at 28/22°C day/night experiment (TTF_COCO), NaCl salt stress experiment (TTF_NACL), mannitol drought stress experiment (TTF_MANN), and abscisic acid 50 uM hormone stress experiment (TTF_ABAT). **(G)** Expression profiles of *I. triloba* NBS-encoding genes in response to various stress: beta-aminobutyric acid biotic stress experiment (TTB_BABA), cold stress at 10/4°C day/night experiment (TTB_COLD), biotic stress control (TTB_BICO), benzothiadiazole S-methylester biotic stress experiment (TTB_BTHT), 6-benzylaminopurine 10 uM hormone stress experiment (TTB_BAPT), hormone control experiment (TTB_HOCO), indole-3-acetic acid 10 uM hormone stress experiment (TTB_IAAT), gibberellic acid 50 uM hormone stress experiment (TTB_GA3T), heat stress at 35/35°C day/night experiment (TTB_HEAT), drought and salt control experiment (TTB_DSCO), heat control at 28/22°C day/night experiment (TTB_HECO), cold control at 28/22°C day/night experiment (TTB_COCO), NaCl salt stress experiment (TTB_NACL), mannitol drought stress experiment (TTB_MANN), and ABA 50 uM hormone stress experiment (TTB_ABAT).

Supplementary Table 1 Primers used in qRT-PCR.

Supplementary Table 2 Nucleotide-binding site (NBS)-encoding genes information of *I. batatas*, *I. trifida*, *I. triloba*, and *I. nil*.

Supplementary Table 3 The motif distribution of CNL-, TNL-, and RNL-type of NBS-encoding genes.

Supplementary Table 4 Organization of nucleotide-binding site (NBS)-encoding genes in the four *Ipomoea* genomes.

Supplementary Table 5 Duplication pattern in sweet potato, *I. trifida*, *I. triloba*, and *I. nil*.

Supplementary Table 6 The syntenic nucleotide-binding site (NBS) gene pairs that are inconsistent with the syntenic chromosomes of the four *Ipomoea* species.

Supplementary Table 7 Nucleotide-binding site (NBS)-encoding orthologous gene groups in the four *Ipomoea* species.

Supplementary Table 8 Ka and Ks of nucleotide-binding site (NBS)-encoding genes within or between sweet potato, *I. trifida*, *I. triloba*, and *I. nil*.
