# Supplementary figures and images for: Genome-wide comparative analysis of the nucleotide-binding site-encoding genes in four Ipomoea species
Source: Front Plant Sci. 2022 Aug 18;13:960723. doi: 10.3389/fpls.2022.960723 (PMC9434374; doi:10.3389/fpls.2022.960723)

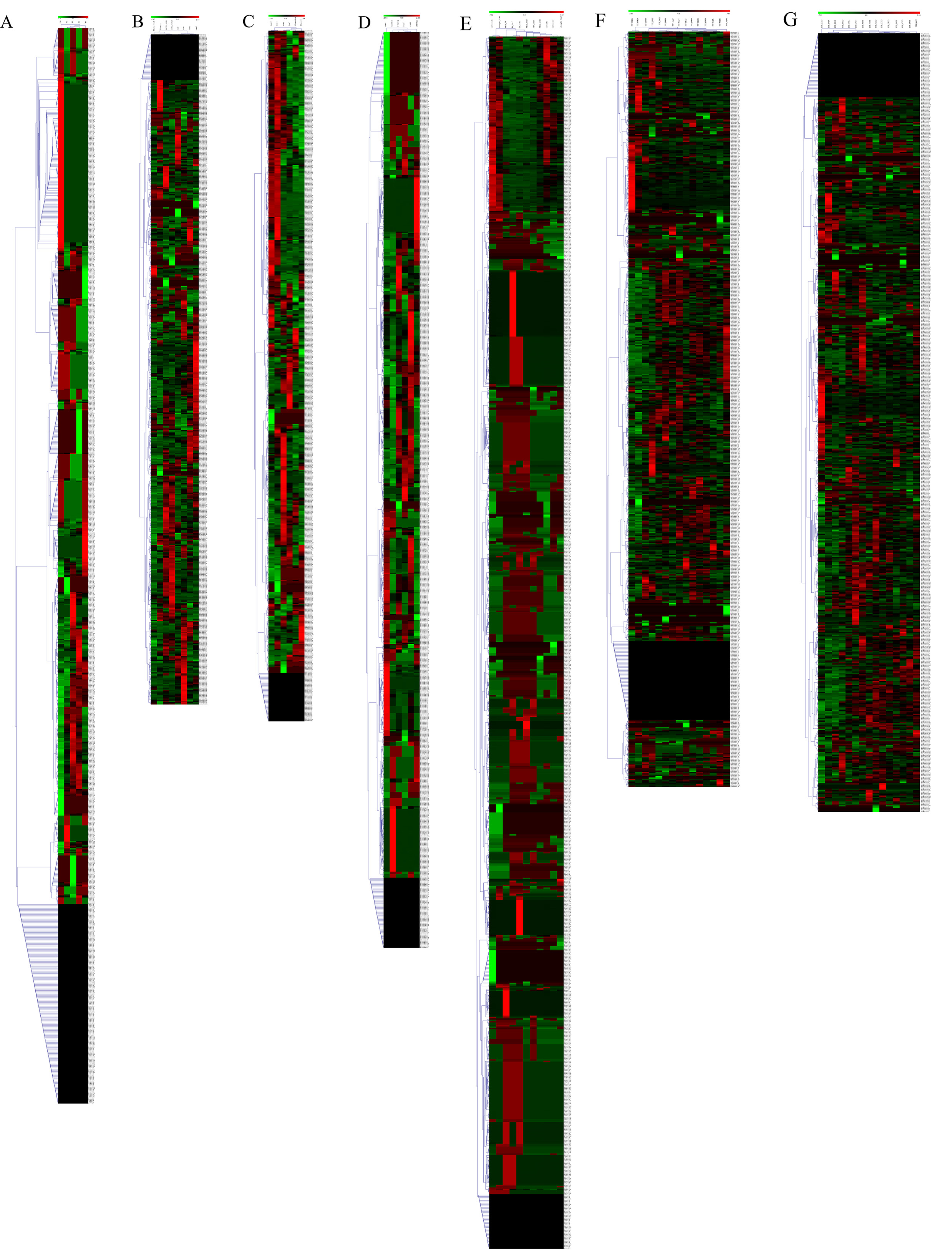

Supplement: Supplementary Figure 1 — Heatmap of the expression profiles of nucleotide-binding site (NBS) genes in the four Ipomoea species. (A) Expression profiles of NBS-encoding genes in different sweet potato tissues: initiative storage root (ISR), distal end (DE), proximal end (PE), root body (RB), and root stalk (RS). (B) Expression profiles of NBS-encoding genes in different I. trifida tissues: the callus flower, callus stem, flower, flower bud, root 1, root 2, leaf, and stem. (C) Expression profiles of NBS-encoding genes in different I. triloba tissues: the root 1, root 2, flower, flower bud, leaf, and stem. (D) Expression profiles of NBS-encoding genes in different I. nil tissues: the root, embryo, seed coat, flower, leaf, and stem. (E) Expression profiles of sweet potato NBS-encoding genes in response to various stress: drought, salt, methyl jasmonate (MeJa), abscisic acid (ABA), and salicylic acid (SA). (F) Expression profiles of I. trifida NBS-encoding genes in response to various stress: beta-aminobutyric acid biotic stress experiment (TTF_BABA), cold stress at 10/4°C day/night experiment (TTF_COLD), biotic stress control (TTF_BICO), benzothiadiazole S-methylester biotic stress experiment (TTF_BTHT), 6-benzylaminopurine 10 uM hormone stress experiment (TTF_BAPT), hormone control experiment (TTF_HOCO), indole-3-acetic acid 10 uM hormone stress experiment (TTF_IAAT), gibberellic acid 50 uM hormone stress experiment (TTF_GA3T), heat stress at 35/35°C day/night experiment (TTF_HEAT), drought and salt control experiment (TTF_DSCO), heat control at 28/22°C day/night experiment (TTF_HECO), cold control at 28/22°C day/night experiment (TTF_COCO), NaCl salt stress experiment (TTF_NACL), mannitol drought stress experiment (TTF_MANN), and abscisic acid 50 uM hormone stress experiment (TTF_ABAT). (G) Expression profiles of I. triloba NBS-encoding genes in response to various stress: beta-aminobutyric acid biotic stress experiment (TTB_BABA), cold stress at 10/4°C day/night experiment (TTB_COLD), biotic [file Image_1.JPEG]
